# Topiramate‐induced acute angle closure with suprachoroidal effusion: Insights from a novel ultra‐wide‐field OCT device

**DOI:** 10.1111/aos.70012

**Published:** 2025-10-01

**Authors:** Michael Hafner, Nicolas Pensel, Nicolas C. D. Scherer, Marc J. Mackert, Siegfried G. Priglinger, Leonie F. Keidel, Maximilian J. Gerhardt

**Affiliations:** ^1^ Department of Ophthalmology LMU University Hospital, Ludwig‐Maximilians‐Universität München Munich Germany

Topiramate‐induced ciliochoroidal effusion syndrome is a rare but potentially vision‐threatening adverse reaction that can precipitate bilateral acute secondary angle‐closure glaucoma. We report two illustrative cases demonstrating the clinical picture, treatment response and characteristic anterior and posterior segment changes using advanced swept‐source ultra‐wide‐field OCT.

## 1 CASE 1

**FIGURE 1:**
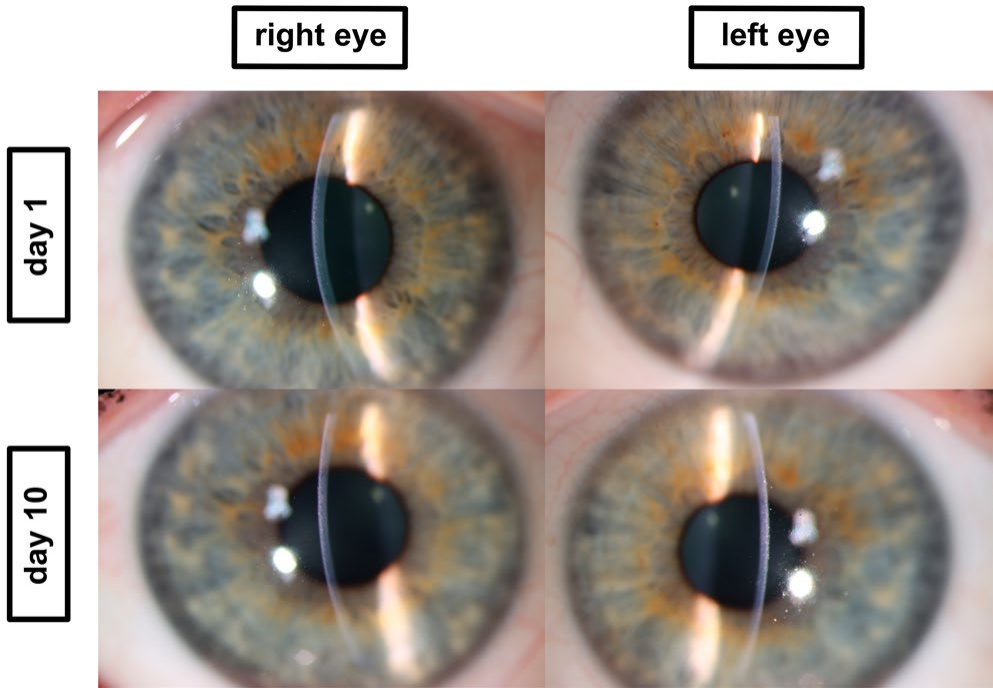
Slit‐lamp anterior segment photographs in Case 1. Top: Both eyes show shallow anterior chambers and mild corneal epithelial oedema. Bottom: After discontinuation of topiramate and initiation of treatment, the anterior chambers deepened and corneal clarity was restored, reflecting complete clinical recovery.

A 35‐year‐old woman presented with bilateral visual impairment, photophobia, ocular discomfort and headache 1 week after starting topiramate for migraine prophylaxis. In the hours prior to presentation, she experienced a sudden worsening of symptoms, with blurred vision, rainbow‐colored halos and a subjective myopic shift. Refraction revealed high myopia (OD −9.00 D, OS −8.25 D). Intraocular pressure (IOP; Goldmann tonometry) was markedly elevated (OD 45 mmHg, OS 48 mmHg). Slit‐lamp examination showed bilateral conjunctival injection, epithelial corneal oedema and extremely shallow anterior chambers (van Herick grade I). Clinical findings are illustrated in Figure 1, which demonstrates anterior chamber shallowing on day 1 with complete resolution by day 10 following drug discontinuation and treatment.

**FIGURE 2:**
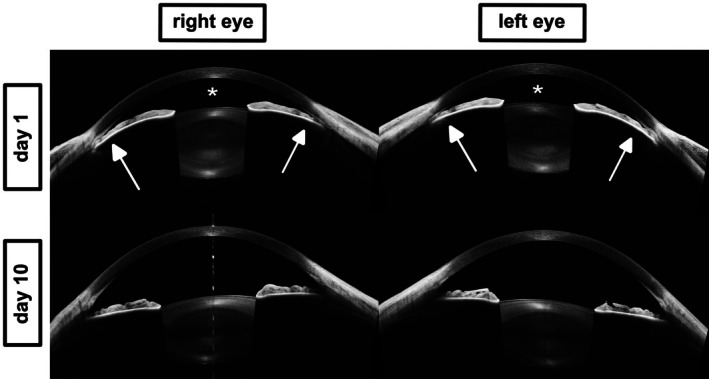
Anterior segment OCT images obtained with the Intalight DREAM system in Case 1. Top: Baseline cross‐sectional OCT images of the right and left eye reveal forward displacement of the iris‐lens diaphragm (white arrows), resulting in marked anterior chamber shallowing (asterisks). Bottom: Follow‐up imaging at day 10 demonstrates complete resolution of these findings and normalization of anterior segment anatomy after drug discontinuation.

**FIGURE 3:**
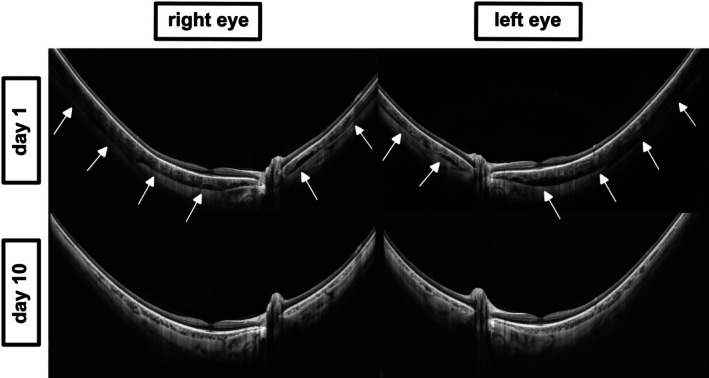
Ultra‐wide‐field OCT imaging of the posterior segment obtained with the Intalight DREAM system in Case 1. Top: Baseline imaging showing bilateral suprachoroidal effusion (white arrows) in the right and left eye on day 1, captured during the acute phase of topiramate‐induced ciliochoroidal effusion syndrome. The images reveal hyporeflective suprachoroidal spaces consistent with fluid accumulation and thickening of the choroid. Bottom: Follow‐up imaging on day 10 after discontinuation of topiramate demonstrates complete resolution of effusion, confirming the reversible nature of this drug‐induced condition.

Anterior segment OCT (AS‐OCT; Intalight® DREAM VG200D) demonstrated anterior displacement of the iris‐lens diaphragm and lens thickening, as shown in Figure 2 (AS‐OCT). Ultra‐wide‐field swept‐source OCT of the posterior segment (Intalight® DREAM VG200D) demonstrated bilateral suprachoroidal effusion, as shown in Figure 3. These are classic signs of topiramate‐induced ciliochoroidal effusion syndrome (Medeiros et al., 2003). Topiramate was discontinued immediately, and the patient was treated with intravenous acetazolamide (500 mg), mannitol (50 g) and topical IOP‐lowering therapy including beta‐blockers, alpha‐agonists, carbonic anhydrase inhibitors and prostaglandin analogs. The patient was admitted and treated in an inpatient setting until IOP had stabilized and the anterior chamber had deepened. Within 24 h, IOP normalized to 15 mmHg in both eyes. Over the following days, the anterior chambers deepened, the myopic shift regressed, and best‐corrected visual acuity improved. By day 10, visual acuity had returned to −0.10 logMAR bilaterally (refraction: OD −5.25 D, OS −4.75 D), and anterior chambers were deep (for clinical image, see Figure 1 bottom panel). AS‐OCT confirmed deepening of the anterior chambers (Figure 2 bottom panel). Follow‐up ultra‐wide‐field OCT confirmed complete resolution of suprachoroidal effusion (Figure 3 bottom panel).

## 2 CASE 2

**FIGURE 4:**
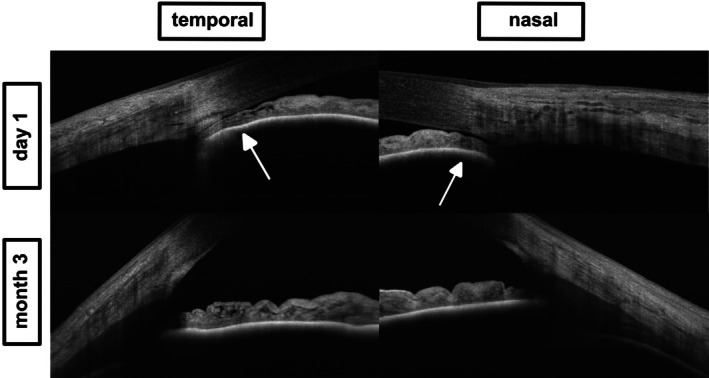
Anterior segment OCT images obtained with the Topcon Triton system in Case 2. Top: Baseline cross‐sectional images acquired on day 1 show bilateral anterior displacement of the iris‐lens diaphragm (white arrows), leading to anterior chamber shallowing. Bottom: Follow‐up imaging at month 3 after discontinuation of topiramate reveals complete anatomical normalization, confirming the reversible nature of the drug‐induced shallowing of the anterior chamber.

**FIGURE 5:**
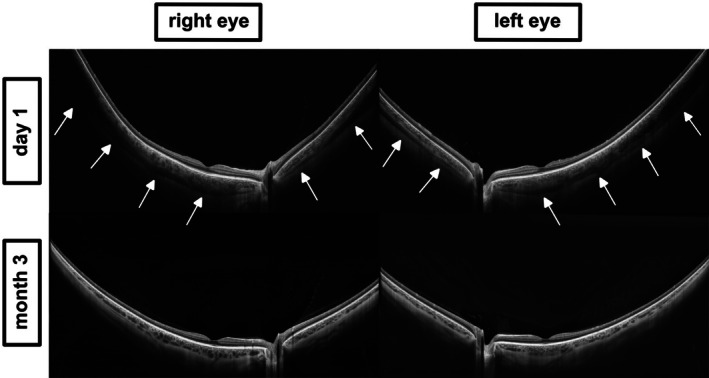
Ultra‐wide‐field OCT images of the posterior segment obtained with the Intalight DREAM system in Case 2. Top: Baseline cross‐sectional scans on day 1 demonstrate bilateral suprachoroidal effusion (white arrows) in both eyes, captured during the acute phase of topiramate‐induced ciliochoroidal effusion syndrome. Hyporeflective spaces between the choroid and sclera are consistent with fluid accumulation. Bottom: Follow‐up imaging at month 3 after discontinuation of topiramate reveals resolution of suprachoroidal effusion.

The second case involved a 30‐year‐old woman who presented with sudden‐onset bilateral vision loss, photophobia and mild headache 12 days after having initiated topiramate therapy for migraine prophylaxis, as in Case 1. Objective refraction showed high myopia (−9.50 D OD, −8.50 D OS), and IOP was elevated at 39 mmHg OD and 38 mmHg OS, respectively. Anterior chambers were shallow (van Herick grade I). Unfortunately, no clinical photograph was available for this case. Corresponding AS‐OCTs are depicted in Figure 4 (taken with Topcon® Triton Swept‐Source OCT). Posterior OCT imaging (with Intalight® DREAM VG200D) revealed bilateral suprachoroidal effusion, analogous to the first case, as shown in Figure 5. The patient was admitted and treated in an inpatient setting until IOP had stabilized and the anterior chamber had deepened. After discontinuation of topiramate and initiation of a similar medical regimen as in case 1, IOP normalized to 11 mmHg in both eyes within 24 h. By day 5, myopic shift had regressed (OD −1.25 D, OS −1.00 D), with visual acuity improving to 0.10 logMAR bilaterally. The anterior chambers had deepened. Follow‐up examination approximately 3 months after initial presentation revealed visual acuity of 0.00 logMAR bilaterally, with an IOP of 14 mmHg in both eyes. At this time point, the anterior and posterior segment OCT demonstrated anatomical resolution (Figures 4 and 5, bottom panels).

These cases underscore the reversible nature of topiramate‐induced secondary angle closure and its distinct pathophysiological mechanism, which differs mechanistically from primary angle‐closure glaucoma. Topiramate is a sulfamated derivative of fructose (Medeiros et al., 2003). Its adverse effects are thought to arise primarily from inhibition of carbonic anhydrase isoenzymes, particularly subtypes II and IV. This inhibition induces ciliary body oedema and ciliochoroidal effusion, causing anterior rotation of the ciliary body and forward displacement of the iris–lens complex. Consequently, the iridocorneal angle closes as well as myopia develops. As a result, neither peripheral iridotomy nor the use of pilocarpine is effective treatment options (Diaz‐Cespedes et al., 2019). Diagnosis is primarily based on the clinical picture (history, medication and clinical examination findings) but is significantly supported by imaging. Similar effects have also been reported for other agents such as, for example, dorzolamide (Fan et al., 1993) as well as sumatriptan (Hsu et al., 2017). Anterior segment OCT and B‐scan ultrasonography are established tools for identifying anterior segment shallowing and choroidal detachments, respectively (Diaz‐Cespedes et al., 2019). More recently, swept‐source ultra‐wide‐field OCT has emerged as a powerful modality for visualizing morphological changes of the posterior segment. In our cases, this imaging technique enabled direct visualization of suprachoroidal effusion, providing anatomical confirmation of the effusive mechanism (see Figures 3 and 5).

To the best of our knowledge, our report is the first to document high‐resolution ultra‐wide‐field OCT findings in this setting. A previous case demonstrated increased choroidal thickness on conventional OCT in a similar patient, supporting the pathophysiological role of suprachoroidal congestion (Vatwani et al., 2023). The ultra‐wide‐field OCT findings presented here offer a more comprehensive visualization of the choroidal changes involved. Early recognition is crucial to prevent irreversible vision loss. These cases emphasize the importance of physician awareness and demonstrate the value of swept‐source ultra‐wide‐field OCT in identifying the pathophysiological hallmark of this condition—suprachoroidal effusion with anterior segment shallowing. Prompt drug discontinuation and IOP‐lowering therapy typically lead to full recovery.

## AUTHOR CONTRIBUTIONS

MH: Conceptualization, Clinical investigation, Imaging acquisition, Imaging analysis, Visualization, Writing: original draft, Writing: review and editing; MJG: Supervision, Clinical investigation, Imaging acquisition, Imaging interpretation, Writing: review and editing; NP: Clinical investigation, Imaging acquisition, Writing: review and editing; LFK: Clinical investigation, Imaging acquisition, Writing: review and editing; MJM: Clinical investigation, Writing: review and editing; NCDS: Clinical investigation, Writing: review and editing; SGP: Supervision, Clinical investigation, Clinical oversight, Writing: review and editing. All authors contributed to the clinical evaluation and management of the patients and approved the final version of the manuscript.

## CONFLICT OF INTEREST STATEMENT

No author reports any financial interests related to this study. However, SGP has received previous speaker fees and/or advisory board honoraria from Novartis Pharma GmbH, Pharm Allergan, Carl Zeiss Meditec AG, BVI, Bayer AG, Alcon Pharma GmbH, Bausch & Lomb (B&L), and Roche AG. SGP also received consulting fees from Roche AG, Carl Zeiss Meditec AG, BVI, and B&L. MJG received speaker fees and/or advisory board honoraria from Bayer, Janssen, Novartis, Rhythm Pharmaceuticals, and Roche. He is a consultant for ViGeneron, with all fees paid to LMU Eye Hospital to support research. LFK received previous speaker fees and/or travel expenses from Novartis Pharma GmbH, Recordati Rare Diseases Inc., CHIESI GmbH, Roche Diagnostics GmbH, DORC Holding BV, and Santen GmbH. MJM reports institutional study support from Implandata, iStar, and Iridex; consulting fees from Santen; honoraria from SightSciences, Abbvie, Santen, and iStar; travel support from Alcon, Abbvie, and iStar; and advisory board participation for Abbvie. He further reports non‐financial support in the form of conference/course sponsorship from the following companies: Abbvie, Zeiss, Optos, Topcon, Polytech Domilens, Alcon, Thea, Ursapharm, Alimera Sciences, Bausch + Lomb, Bayer Vital, Novartis, OmniVision, Rayner, Roche Pharma, Heidelberg Engineering, Santen, Thea Pharma, TRB, Vanda Pharmaceuticals, and Sight Sciences. MH, NP, and NCDS declare no financial disclosures. All authors confirm that there are no conflicts of interest regarding this manuscript. An intramural funding was granted to MJG and LFK (Munich Medical & Clinician Scientist Program, LMU Munich). The funder played no role in study design, data collection, analysis, and interpretation of the data or the writing of this manuscript.

## Data Availability

The datasets used and analysed during the current study are available from the corresponding author upon reasonable request, subject to institutional data sharing agreements and participant consent provisions.
